# Nonsurgical management of photoaversive ocular and systemic loiasis in Michigan

**DOI:** 10.3205/oc000149

**Published:** 2020-04-15

**Authors:** Sneha Padidam, Hamilton Trinh, Xihui Lin, Joseph D. Boss

**Affiliations:** 1 Kresge Eye Institute/Wayne State University School of Medicine, Detroit, USA; 2 Wayne State University School of Medicine, Detroit, USA; 3 Cole Eye Institute, Cleveland Clinic, Cleveland, USA

**Keywords:** ocular loiasis, Loa loa nematode, apheresis

## Abstract

**Objective:** Ocular loasis refers to ocular conditions such as pain and redness caused by the movement of the *Loa loa* nematode through the subconjuctival space of the eye. It is a tropical disease that is very rarely seen in North America. We report the case of a 32-year-old male who was recently diagnosed with ocular loasis in the Midwestern region of the United States.

**Methods:** He presented to the emergency department with left eye pain after seeing a “worm in his eye” the previous night. He had emigrated from Cameroon 7 years prior. Anterior segment examination revealed a translucent, motile worm in the subconjunctival space of his left eye.

**Results:** Prior to the patient’s scheduled follow-up for surgical removal of the worm, it migrated into the lower eyelid subdermal space. Serum testing confirmed the presence of *Loa loa* microfilariae at a concentration of >17,000 mf/mL.

**Conclusion:** The patient was treated at the National Institute of Health (NIH) with pheresis followed by diethylcarbamazine and reported symptomatic improvement 1 month after treatment. This case report demonstrates the importance of being able to recognize and properly manage vector-borne parasites in nonendemic areas due to increased travel and climate change.

## Introduction

The combination of increased global travel and climate change has increased the presence of vector-borne parasites in more northern regions of the United States [1]. Clinicians in non-endemic regions must be well-versed in parasitic infections, now more than ever. *Loa loa*, one of the nine nematodes that use humans as a host, infects an estimated 12 million people in West and Central Africa but is rarely seen in the United States or Europe [2]. Ocular loiasis can occur as the nematode migrates into the adnexae or subconjunctival space, presenting with ocular pain, redness, foreign body sensation, in addition to an often-visible motile nematode in the subconjunctival space.

## Case description

**Figure 1:**
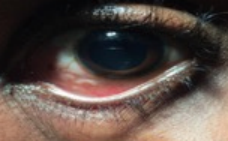
Left eye shows presence of curvilinear nematode in the inferonasal subconjunctival space.

A 32-year-old man presented to a Michigan emergency department with left eye pain after seeing a “worm in his eye” the previous night. He had emigrated from Cameroon 7 years prior. He had no prior medical or ocular history. On examination, the left eye had mild conjunctival injection and chemosis. A thin, translucent, motile nematode was visualized moving in the sub-bulbar conjunctival space (Figure 1 (Fig. 1)). The visual acuity in both eyes was 20/20. No intraocular or contralateral inflammation or sequelae were present. Systemic examination did not reveal any abnormalities. Blood count showed 14% eosinophils (range 0–8). A blood smear was obtained and showed microfilariae compatible with *Loa loa*. Serum was sent to the NIH for further testing and later revealed that the patient was highly microfilaremic (>17,000 mf/mL) with *Loa loa* but negative for OV16 and Wb123, ruling out *Onchocerca volvulus* and *Wuchereriabancrofti*.

At the time of surgical extraction the following day in the eye clinic, the nematode’s photoaversion to the microscope light caused it to migrate out of view of the sub-bulbar conjunctiva posteriorly and later to the sub-palpebral conjunctival space. Due to the inability to isolate the nematode under direct visualization after multiple attempts, the decision was made to treat with systemic medications. Given the high serum microfilariae load with concern for post-treatment encephalitis, the patient was treated at the NIH with pheresis followed by diethylcarbamazine. One month post-therapy, the patient was asymptomatic.

## Discussion

Loiasis is a systemic parasitic infection caused by the *Loa loa* nematode. It is endemic to West and Central Africa, including Cameroon, from which our patient emigrated 7 years prior. Given the long lifespan of the adult worm (average life expectancy of 9 years) as well as continuous exposure to repeat infection, individuals in endemic regions may harbor the infection for their entire lives [3]. On the other hand, ocular loiasis is very rare in non-endemic nations. Per Antinori et al., there have been 46 cases of ocular loiasis outside of endemic regions and only 9 reported cases within the United States between 1986 and 2011 [4]. One case reported in the United States since 2011 is of a 27-year-old male also from Cameroon [2]. *Loa loa* is transmitted to humans through transmission of microfilariae from the bite of an infected Chrysops fly. Adult nematodescan live in human hosts for up to 17 years [5]. While most cases of loiasis are asymptomatic, patients can experience swelling of the subcutaneous tissues known as Calabar swellings caused by the mature nematodes. Our patient admitted to an episode of unexplained swelling of his right forearm years ago. A definitive sign of infection is visualization of the nematode in the subconjunctival space of the eye [6]. While this is the most common ocular presentation, there are case reports of the adult nematodes in the anterior chamber, eyelid, and even in the vitreous cavity [7], [8], [9]. Rarer systemic signs associated with loiasis include endo-myocardial fibrosis, pulmonary infiltrates, and renal failure. These signs are attributed to immune complex deposition, are associated with eosinophilia, and are more commonly seen in endemic regions [5]. *Loa loa* can also be diagnosed by the presence of microfilariae on peripheral blood smear, although false negatives can occur.

Removal of the nematode from the eye is completed with paralysis of the nematode with local anesthetic and surgical removal from the subconjunctival space [1], [10]. This relieves pain and allows confirmation of diagnosis through histological exam, but is not essential. In our case, the *Loa loa* nematode became photoaversive at numerous attempted surgical extraction attempts, migrating posteriorly out of view within the sub-conjunctival plane. Even in cases when the nematode is successfully removed, systemic antimicrobial treatment is a necessity for a cure. Diethylcarbamazine is the World Health Organization’s recommended first-line treatment for systemic loaisis. Ivermectin and albendazole are second-line treatments. When the microfilarial load is greater than >8,000 mf/mL as in our patient, there exists a risk of encephalitis with treatment, due to lysis of the microfilariae resulting in an inflammatory response [5]. Pheresis to remove microfilariae in the buffy coat has been reported as a successful method in reducing the microfilarial load to prevent encephalitis prior to actual systemic diethylcarbamazine treatment [11], [12], [13].

It is important to note, however, that pheresis may not be readily available in many endemic regions [14]. Since loiasis has often been regarded as benign, it is often not treated in endemic areas due to the lack of resources as well as the increased risk of encephalitis associated with the lysis of a high microfilarial load [3], [14]. Patients in endemic areas who are infected with onchocerciasis or lymphatic filiarasis are often co-infected with *Loa loa*. This co-infection with *Loa loa* complicates mass treatment of onchocerciasis or lymphatic filiarasis with ivermectin due to the increased risk of the systemic inflammatory response associated with the lysis of a high filarial load [15], [16]. A 2007 study out of Cameroon involving over 4,000 respondents noted the prevalence of *Loa loa* microfileremia to be >20% in most of the study villages [17]. These patients are at risk of possible systemic complications of loasis including endomyocardial fibrosis, pulmonary infiltrates and renal failure [5]. Research to improve diagnosis and therapy of loasis as well as to elucidate its effect on affected populations is needed.

Given the increased presence of vector-borne parasites in non-endemic regions, it is imperative that all physicians maintain a high index of suspicion of this clinical entity especially when travel or immigration history point to exposure. Knowledge of nonsurgical management options of ocular loiasis, when surgical extraction is unachievable due to lack of access to trained ocular surgeons, proper equipment, or due to the photoaversive nature of the organism, as seen in our case, is crucial. 

## Notes

### Competing interests

The authors declare that they have no competing interests.
